# From Hesitation to Confidence: Shifting Parental Attitude and Perception Toward Ketogenic Diet in Treating Children With Drug-Resistant Epilepsy

**DOI:** 10.7759/cureus.81408

**Published:** 2025-03-29

**Authors:** Soma Basu, A J Hemamalini, Ranjith Kumar Manokaran

**Affiliations:** 1 Department of Clinical Nutrition, Faculty of Allied Health Sciences, Sri Ramachandra Institute of Higher Education and Research, Chennai, IND; 2 Department of Neurology, Sri Ramachandra Institute of Higher Education and Research, Chennai, IND

**Keywords:** dietary adherence, ketogenic diet, parental perceptions, pediatric epilepsy therapy, refractory epilepsy, structured intervention

## Abstract

Background: Managing drug-resistant epilepsy (DRE) in children through ketogenic diets (KD) is a promising therapeutic approach. Parental perceptions significantly influence adherence and outcomes of KD; however, to date, no validated tool exists to evaluate these perceptions systematically. This study presents a novel approach by developing and validating the first questionnaire to assess parental perceptions of KD in a clinical setting. The aim of this study was to develop and evaluate changes in parental perceptions and attitudes toward KD before and after a structured intervention, addressing domains such as awareness, perceived effectiveness, compliance, concerns, and social and financial influences.

Methodology: A pre- and post-intervention study was conducted among 30 parents of children with drug-resistant epilepsy aged one month to five years. Structured educational sessions, counseling, and resource distribution were provided over three months. Parental perceptions were assessed using validated questionnaires at baseline and post-intervention. The internal consistency of the domains was analyzed using Cronbach's alpha, with values ≥0.70 deemed acceptable. Paired t-tests compared pre- and post-intervention scores, with statistical significance set at p < 0.05. The developed questionnaire has been duly registered by the Copyright Office of the Government of India and is copyright-protected. The study was approved by the Institutional Ethics Committee of Sri Ramachandra Institute of Higher Education and Research, Porur, Chennai (reference number: IEC/24MAR/185/09).

Results: Post-intervention, significant improvements were observed in parental understanding (mean: 5.000 ± 0.000 versus 2.167 ± 1.02; p < 0.001), perceived effectiveness (4.700 ± 0.915 versus 2.967 ± 0.513; p < 0.001), and motivation (4.533 ± 0.698 versus 3.267 ± 0.521; p < 0.001). Concerns about side effects, financial burden, and long-term adherence decreased significantly (p < 0.001). Cronbach's alpha demonstrated high internal consistency across domains (α ≥ 0.719). Positive impacts on child quality of life and reduced peer influence were also noted.

Conclusion: Structured education and support significantly improved parental perceptions, alleviating barriers to KD adherence. The validated questionnaire serves as a robust clinical tool for assessing and addressing parental perceptions, enabling tailored interventions to enhance KD efficacy and optimize outcomes in children with refractory epilepsy.

## Introduction

Parental perceptions and experiences play a critical role in the implementation of therapeutic interventions such as ketogenic diets (KD) for managing drug-resistant epilepsy in children. The KD, a high-fat, low-carbohydrate dietary regimen, has been extensively studied for its efficacy in reducing seizure frequency, particularly in children with drug-resistant epilepsy (DRE) [1,2]. Research has consistently demonstrated that adherence to KD protocols is crucial for achieving optimal seizure control, with parental commitment serving as a key determinant of compliance and long-term success [3].

Despite the robust evidence supporting KD efficacy, several barriers hinder its sustained implementation. Studies have identified factors such as limited awareness, misconceptions about the diet, and concerns regarding its potential side effects as common challenges faced by families [4,5]. Psychosocial factors, including family dynamics, cultural beliefs, and financial constraints, further influence parental attitudes toward KD, often impacting adherence and treatment outcomes [6,7].

Parental understanding, motivation, and perception of the intervention's effectiveness are pivotal in ensuring long-term compliance and maximizing benefits for the child's quality of life. Positive parental perceptions have been linked to improved dietary adherence, reduced caregiver burden, and enhanced seizure control outcomes [8]. Conversely, inadequate counseling and insufficient parental support may contribute to premature diet discontinuation or poor compliance, ultimately affecting treatment efficacy [9].

To date, no validated tool systematically evaluates parental perceptions of ketogenic diets (KD) in managing pediatric drug-resistant epilepsy. This study introduces a novel approach by designing and validating the first questionnaire to assess parental perceptions of ketogenic diets (KD) in managing pediatric drug-resistant epilepsy in clinical settings. The objective was to assess and measure changes in parental perceptions and attitudes toward KD before and after a structured intervention. Key domains addressed included awareness, perceived effectiveness, compliance, concerns about side effects, and social and financial influences. This innovative tool provides a comprehensive framework to understand and address parental experiences, ultimately enhancing adherence and optimizing outcomes in children with drug-resistant epilepsy.

## Materials and methods

Study design and population

A pre- and post-intervention study design was adopted to evaluate changes in parental perceptions across multiple domains. Participants included 30 parents of children with drug-resistant epilepsy who were assigned to undergo ketogenic dietary therapy by the pediatric epileptologist.

Ethical consideration

The study was approved by the Institutional Ethics Committee of Sri Ramachandra Institute of Higher Education and Research, Porur, Chennai (reference number: IEC/24MAR/185/09).

Copyright statement

The developed questionnaire has been duly registered by the Copyright Office of the Government of India and is copyright-protected.

Type of sampling and reasons for selection

The questionnaire was developed to assess various factors influencing parental attitudes, with domains such as awareness, perceived effectiveness, dietary management, quality of life, and external influences. Eligible participants were recruited from a tertiary care center by the pediatric epileptologist.

Patient consent statement

The inclusion of this study was in line with the Declaration of Helsinki. Parental consent was taken before enrolling the participants. All potential participants' parents were informed about the research goals. Parents were assured that no adverse effects would be anticipated from their child's participation in the current research. Furthermore, their data were guaranteed to be anonymous and confidential, and their collaboration was voluntary. The participants were included in the study after obtaining parental consent.

Inclusion criteria

The inclusion criteria were parents of children with confirmed drug-resistant epilepsy aged one month to five years and willingness to participate in a ketogenic dietary regimen and complete pre- and post-intervention questionnaires.

Exclusion criteria

We excluded parents of children who are above five years of age.

Data collection method

The intervention comprised structured educational sessions focusing on the principles of ketogenic diets, their therapeutic role in epilepsy, management strategies, and addressing parental concerns. In addition, ongoing support through counseling sessions and resource distribution was provided over three months. Data were collected using validated questionnaires administered at baseline (pre-intervention) and three months post-intervention. Domains assessed included parental understanding and awareness, perceived effectiveness, dietary management and compliance, concerns and side effects, support and resources, parental motivation and decision-making, impact on the child's quality of life, long-term considerations, community and peer influence, and financial considerations.

Statistical analyses

The internal consistency of the questionnaire domains was verified using Cronbach's alpha, with values ≥ 0.70 considered acceptable. Descriptive statistics were used to summarize baseline characteristics. Paired t-tests were performed to compare mean scores of parental perceptions pre- and post-intervention across each domain. A p-value < 0.05 was considered statistically significant. Cronbach's alpha was calculated to assess the reliability of the domains within the questionnaires.

## Results

A comparison of means of parental perceptions pre- and post-intervention relevant to each domain in the two questionnaires using independent t-tests is presented in Table 1.

**Table 1:** Comparison of the Means of Parental Perceptions Pre- and Post- Intervention on the Domains Tested Using the Questionnaires SD: standard deviation

Domains	Mean ± SD		T	P
	Pre-intervention	Post-intervention		
Parental understanding and awareness	2.167 ± 1.02	5.000 ± 0.000	-15.216	0.000
Perceived effectiveness	2.967 ± 0.513	4.700 ± 0.915	-9.047	0.000
Dietary management and compliance	3.500 ± 0.313	2.733 ± 0.450	7.667	0.000
Concerns and side effects	3.300 ± 0.237	2.467 ± 0.166	15.757	0.000
Support and resources	3.400 ± 0.603	4.767 ± 0.341	-10.812	0.000
Parental motivation and decision-making	3.267 ± 0.521	4.533 ± 0.698	-7.965	0.000
Impact on the child's quality of life	3.500 ± 0.347	3.733 ± 0.423	-2.333	0.023
Long-term considerations	3.933 ± 0.254	3.267 ± 0.365	8.212	0.000
Community and peer influence	3.250 ± 0.341	2.625 ± 0.346	7.047	0.000
Financial considerations	3.067 ± 0.563	1.667 ± 0.429	10.832	0.000

From the results, it can be observed that the opinions of parents concerning epileptic children taking up a ketogenic diet varied significantly before and after the intervention, as indicated by a p-value of less than 0.05 in all the cases. The parental understanding and awareness of ketogenic diets were found to increase radically post-intervention (5.000 ± 0.000) compared to pre-intervention (2.167 ± 1.02) (t = -15.216, p = 0.000). Similarly, the parents' perceived effectiveness of ketogenic diets for epileptic children profoundly increased post-intervention (4.700 ± 0.915) as against pre-intervention (2.967 ± 0.513) (t = -9.047, p < 0.000). There was an increase in the support and resources post-intervention (4.767 ± 0.341) than pre-intervention (3.400 ± 0.603) (t = 10.812, p = 0.000). Likewise, parental motivation and decision-making regarding ketogenic diets significantly showed improvements post-intervention (4.533 ± 0.698) compared to pre-intervention (3.267 ± 0.521) (t = -7.965, p = 0.000). Subsequently, post-intervention, there was a statistically positive impact on the child's quality of life (3.733 ± 0.423) over pre-intervention (3.500 ± 0.347) (t = -2.333, p = 0.023). In contrast, the parents' queries about future dietary management and compliance issues were less post-intervention (2.733 ± 0.450) compared to pre-intervention (3.500 ± 0.313) (t = 7.667, p = 0.000). Pre-conceived concerns on side effects related to ketogenic diets were less post-intervention (2.467 ± 0.166) than pre-intervention (3.300 ± 0.237) (t = 15.757, p = 0.000). The parents' hesitation related to the adoption of ketogenic diets for the long term also decreased post-intervention (3.267 ± 0.365) compared to pre-intervention (3.933 ± 0.254) (t = 8.212, p = 0.000). Community and peer influence were all found to be minimized post-intervention (2.625 ± 0.346) over pre-intervention (3.250 ± 0.341) (t = 7.047, p = 0.000). Lastly, the apprehension on financial considerations was significantly lowered among parents post-intervention (1.667 ± 0.429) compared to pre-intervention (3.067 ± 0.563) (t = 10.832, p = 0.000).

Table 2 presents the reliability of the domains used in the questionnaire, assessed by measuring internal consistency using Cronbach's alpha. Generally, a value of 0.70 or higher is considered acceptable. It was found that all the domains considered for the study had an acceptable internal consistency. Excellent consistency was observed among parental understanding and awareness (α = 0.981; three items), perceived effectiveness (α = 0.941; three items), impact on the child's quality of life (α = 0.965; two items), concerns and side effects (α = 0.915; two items), and financial considerations (α = 0.901; two items). A good level of consistency existed among parental motivation and decision-making (α = 0.885; three items), support and resources (α = 0.861; three items), community and peer influence (α = 0.894; two items), and dietary management and compliance (α = 0.841; two items), while an acceptable level of consistency existed in long-term considerations (α = 0.719; two items).

**Table 2:** Reliability Tests This table presents the reliability of the domains used in the questionnaire, assessed by measuring internal consistency using Cronbach's alpha. Generally, a value of 0.70 or higher is considered acceptable.

Factors	Cronbach's alpha	Number of items
Parental understanding and awareness	0.981	3
Perceived effectiveness	0.941	3
Dietary management and compliance	0.841	2
Concerns and side effects	0.915	2
Support and resources	0.861	3
Parental motivation and decision-making	0.885	3
Impact on the child's quality of life	0.965	2
Long-term considerations	0.719	2
Community and peer influence	0.894	2
Financial considerations	0.901	2

## Discussion

The findings of this study underscore the significant impact of structured interventions on parental perceptions and attitudes toward ketogenic dietary (KD) therapy in managing drug-resistant epilepsy. Pre-intervention results revealed gaps in parental understanding, perceived efficacy, and concerns regarding dietary adherence, long-term implications, and financial constraints. Post-intervention, these perceptions shifted positively, highlighting the value of educational and counseling support.

The ketogenic diet (KD) differs significantly from a regular diet, primarily due to its macronutrient composition, which emphasizes high fat, low carbohydrate, and moderate protein intake. This shift in dietary pattern induces a state of ketosis, where the body relies on fat-derived ketones for energy instead of glucose. However, the adoption of KD presents several challenges, particularly due to its unconventional nature. The high-fat, low-carbohydrate requirements can make it difficult for individuals, especially children, to adapt, as it often limits familiar or culturally preferred food options. For older children, this dietary restriction may further narrow their already limited preferences, leading to issues with compliance. Parental concerns also play a significant role, particularly regarding nutritional adequacy, taste acceptance, and the overall impact on the child's growth and development. The diet's taste and texture, which differ from conventional meals, may present sensory challenges for children, reducing palatability and increasing resistance. Financial considerations also arise, as KD often necessitates specific, high-quality ingredients and professional guidance, potentially making it less accessible for families with limited resources. General challenges reported with KD include limited food choices, social isolation during meal times, and the need for meticulous meal planning and preparation.

Despite these barriers, expert education and targeted support can significantly enhance acceptance and adherence to the diet. Professional guidance from dietitians, neurologists, and support teams can demystify the complexities of KD by educating parents and caregivers about its nutritional benefits, meal preparation, and management strategies. This education helps to alleviate common concerns, such as whether the diet meets the child's nutritional needs or affects their growth. Experts can also provide tailored solutions to improve palatability, such as creating appealing recipes and incorporating culturally familiar ingredients, thereby enhancing the diet's acceptability among children. Addressing practical challenges, such as meal planning and affordability, further promotes adherence. By equipping families with cost-effective strategies and resources to source appropriate foods, professionals can make the diet more accessible. Additionally, providing emotional support and connecting families with peer networks or communities can help reduce feelings of isolation, increasing long-term commitment to the dietary regimen. For children with epilepsy, such targeted interventions are transformative. KD has been shown to significantly reduce seizure frequency and, in some cases, achieve seizure freedom. Improved adherence through expert support maximizes these therapeutic outcomes, contributing to better neurological and developmental health. Furthermore, enhancing family confidence in managing the diet empowers caregivers and improves the child's overall quality of life. Thus, expert education and addressing parental concerns are pivotal in overcoming the challenges associated with KD. By facilitating acceptance and adherence, professionals can ensure that children with epilepsy fully benefit from this evidence-based dietary intervention, leading to meaningful improvements in their health and well-being.

Parental understanding and awareness

The intervention notably improved parental awareness (mean pre-intervention: 2.167 ± 1.02, post-intervention: 5.000 ± 0.000, p < 0.0001). This emphasizes that structured education sessions addressing the principles and therapeutic roles of KD significantly enhance knowledge. Previous research has indicated that knowledge gaps can impede adherence to dietary therapies, making awareness-building a cornerstone for success in KD interventions [10,11].

Perceived effectiveness and motivation

Parental perceptions of KD effectiveness improved substantially (mean pre-intervention: 2.967 ± 0.513, post-intervention: 4.700 ± 0.915, p < 0.0001). The motivation for long-term adherence also increased (mean pre-intervention: 3.267 ± 0.521, post-intervention: 4.533 ± 0.698, p < 0.0001). These findings align with studies demonstrating that belief in therapeutic efficacy correlates positively with adherence and willingness to maintain interventions over time [12,13].

Addressing concerns and barriers

Concerns regarding side effects, dietary compliance, and financial burdens were significantly alleviated post-intervention. Financial apprehensions saw the most dramatic decline (mean pre-intervention: 3.067 ± 0.563, post-intervention: 1.667 ± 0.429, p < 0.0001), highlighting the importance of addressing economic factors. Similarly, perceived side effects decreased (mean pre-intervention: 3.300 ± 0.237, post-intervention: 2.467 ± 0.166, p < 0.0001), suggesting that reassurance and consistent follow-up can mitigate fears, as observed in prior research [14].

Quality of life and social influences

There was a statistically significant improvement in parental perceptions of the child's quality of life (mean pre-intervention: 3.500 ± 0.347, post-intervention: 3.733 ± 0.423, p = 0.023). This aligns with existing evidence that effective epilepsy management through KD can enhance overall family well-being. Community and peer influence also showed significant reductions, indicating that empowering parents with accurate knowledge and resources may counteract external skepticism [15,16].

Implications for practice

The results emphasize the importance of incorporating structured support mechanisms into KD therapy. Educational sessions, ongoing counseling, and resource distribution not only improve adherence but also positively influence parental perceptions across multiple domains. These findings advocate for a multidisciplinary approach that includes dietitians and neurologists to optimize therapeutic outcomes.

Strength of the study

This study is the first to develop and validate a systematic tool to assess parental perceptions of the ketogenic diet (KD), filling a crucial gap in pediatric epilepsy management. The study implemented a well-designed educational and counseling program, ensuring standardized delivery of information and support to parents. The use of a validated assessment tool with high internal consistency (Cronbach’s alpha ≥ 0.719) enhances the reliability of the findings. The study employed pre- and post-intervention assessments, allowing for a quantitative evaluation of changes in parental attitudes and concerns. By addressing parental concerns, the study highlights an effective approach to improving dietary adherence and optimizing therapeutic outcomes in children with drug-resistant epilepsy.

Limitation of the study

The study was conducted among 30 parents, which limits the generalizability of the findings to larger populations. The intervention lasted three months, making it difficult to assess the long-term sustainability of improved perceptions and adherence.

## Conclusions

The study demonstrates that structured interventions significantly improved parental perceptions of ketogenic dietary therapy for managing drug-resistant epilepsy. The three-month intervention comprised structured educational sessions focusing on the principles of ketogenic diets, their therapeutic role in epilepsy, and management strategies and addressing parental concerns. In addition, ongoing support through counseling sessions and resource distribution was provided over three months. Post-intervention, parents exhibited enhanced understanding, motivation, and perceived effectiveness, alongside reduced concerns about side effects, financial constraints, and social influences. These findings highlight the critical role of education and support in promoting adherence and optimizing therapeutic outcomes.

## Figures and Tables

**Figure 1:**
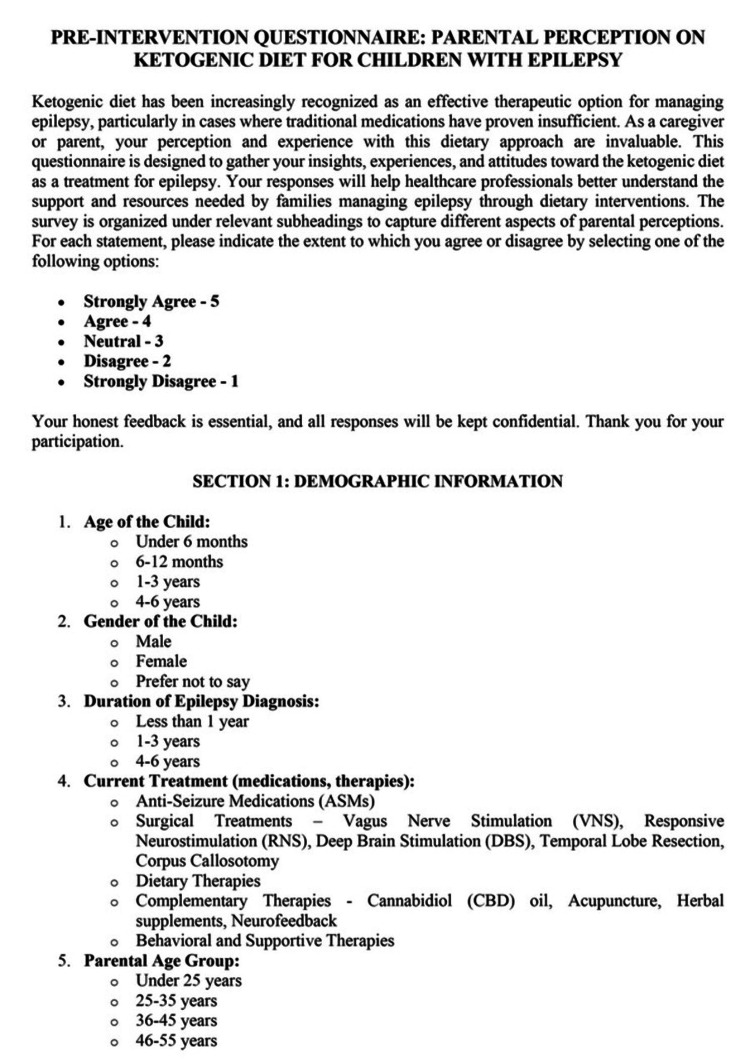
Questionnaire Page 1

**Figure 2:**
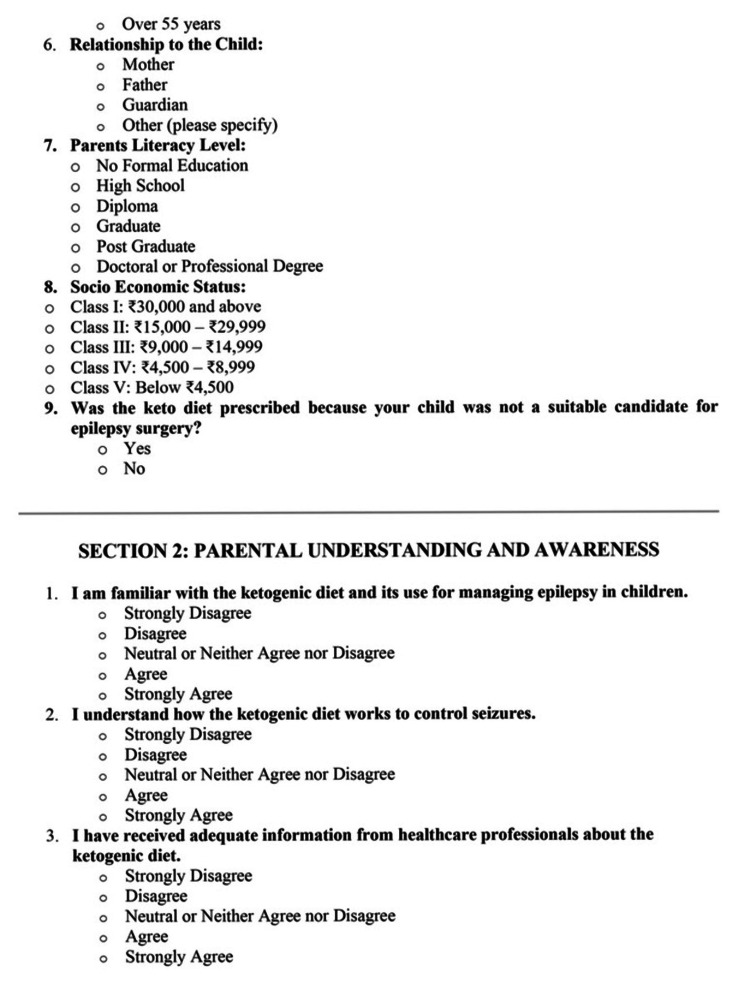
Questionnaire Page 2

**Figure 3:**
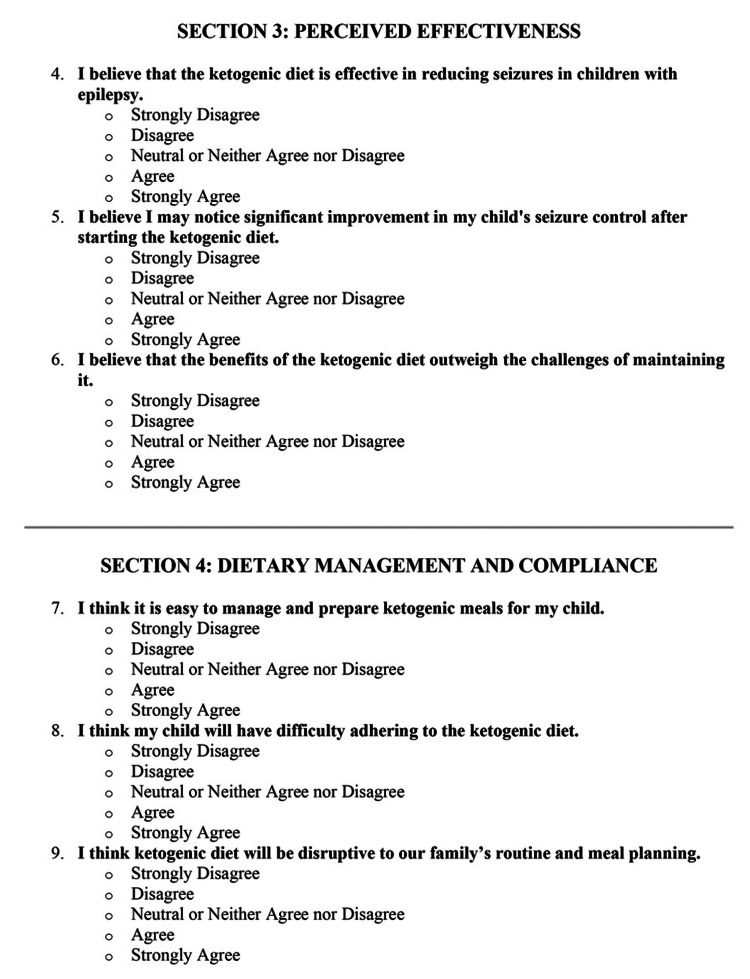
Questionnaire Page 3

**Figure 4:**
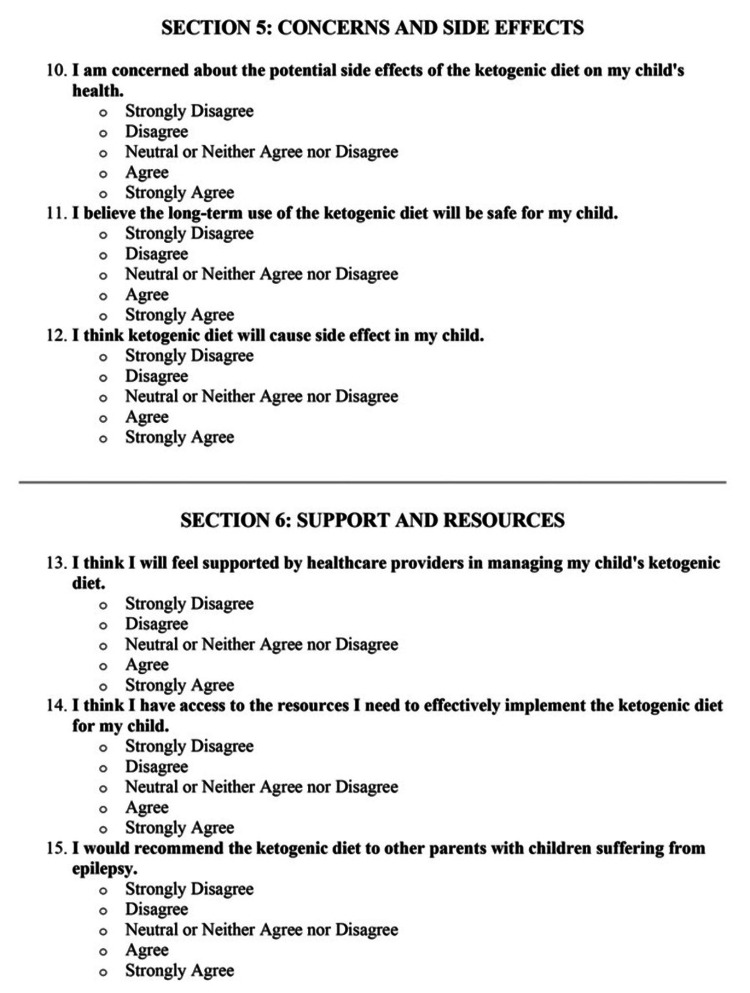
Questionnaire Page 4

**Figure 5:**
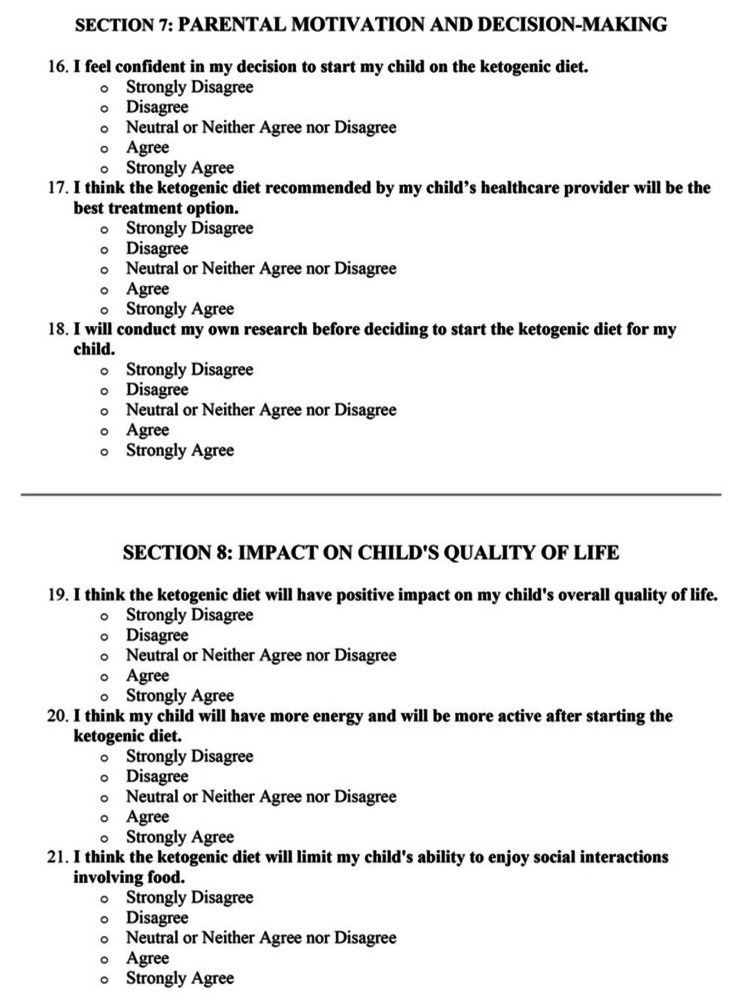
Questionnaire Page 5

**Figure 6:**
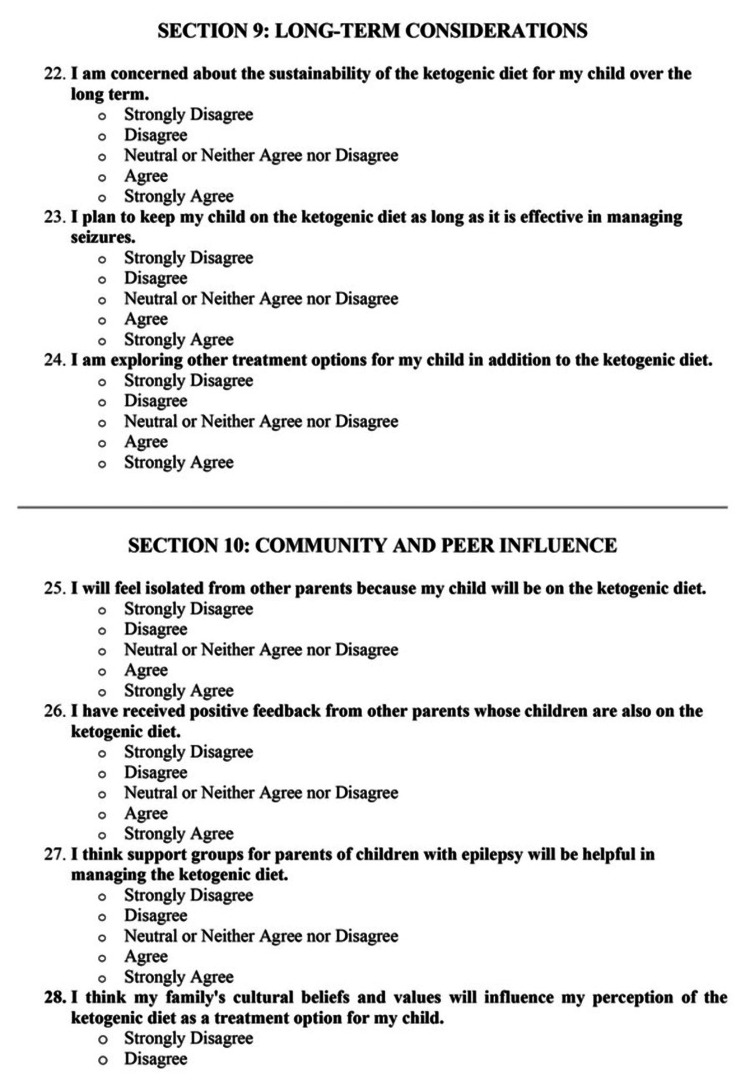
Questionnaire Page 6

**Figure 7:**
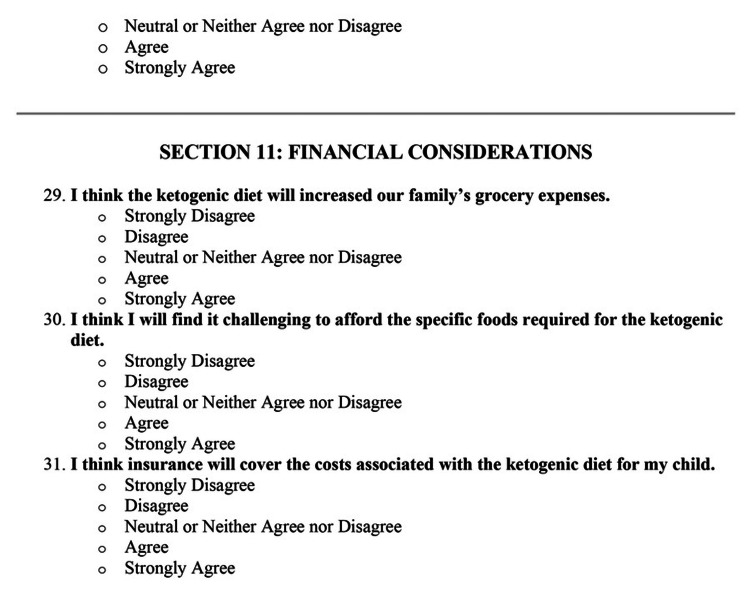
Questionnaire Page 7

**Figure 8:**
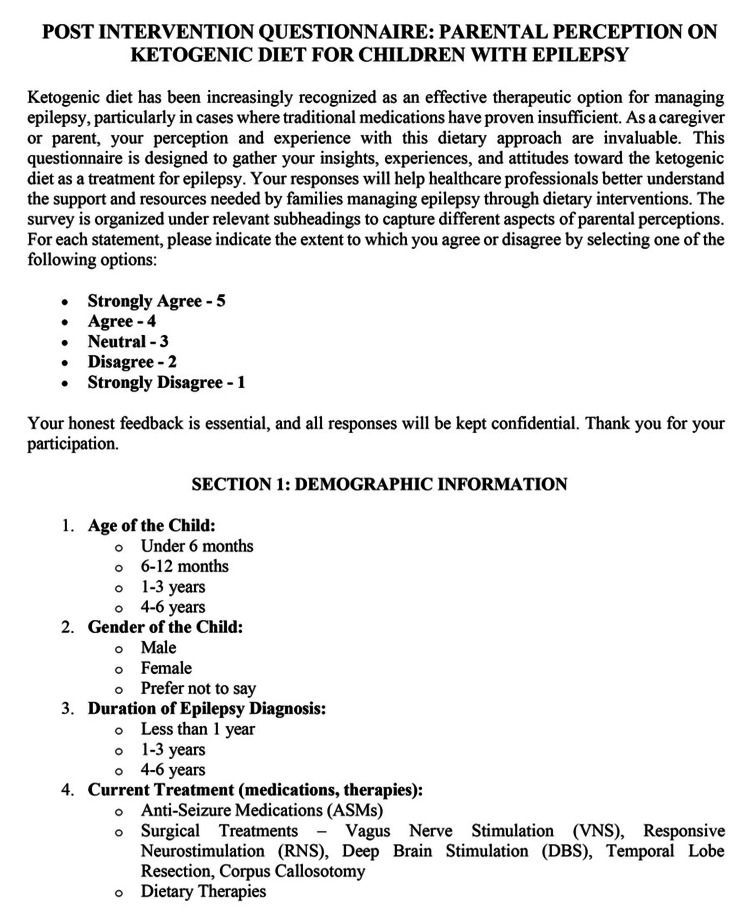
Questionnaire Page 8

**Figure 9:**
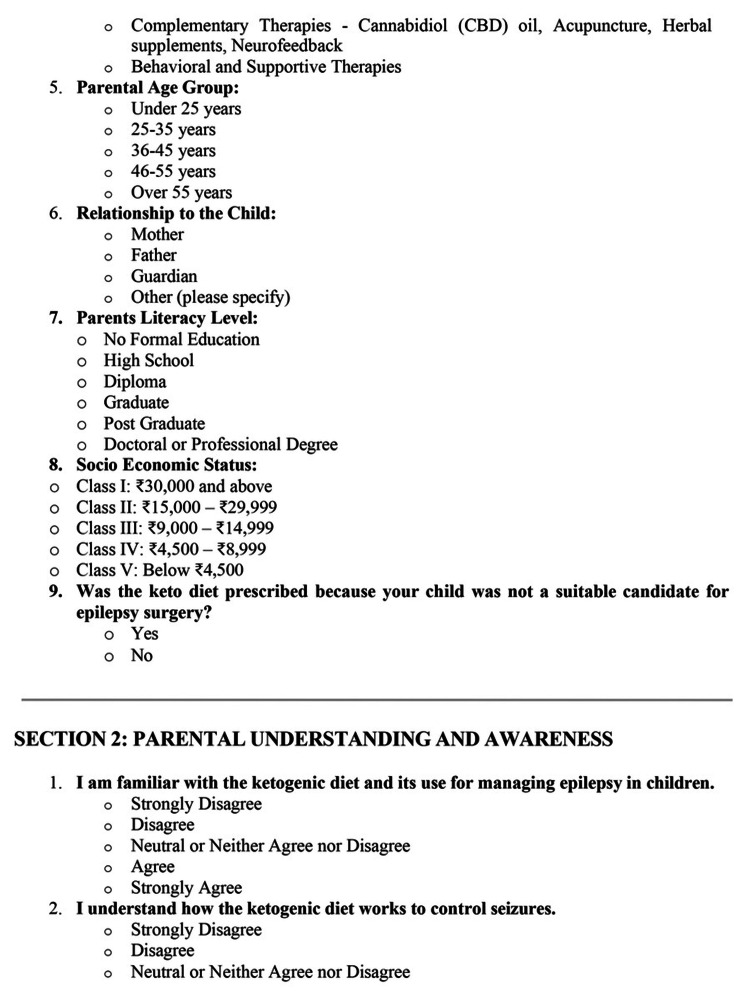
Questionnaire Page 9

**Figure 10:**
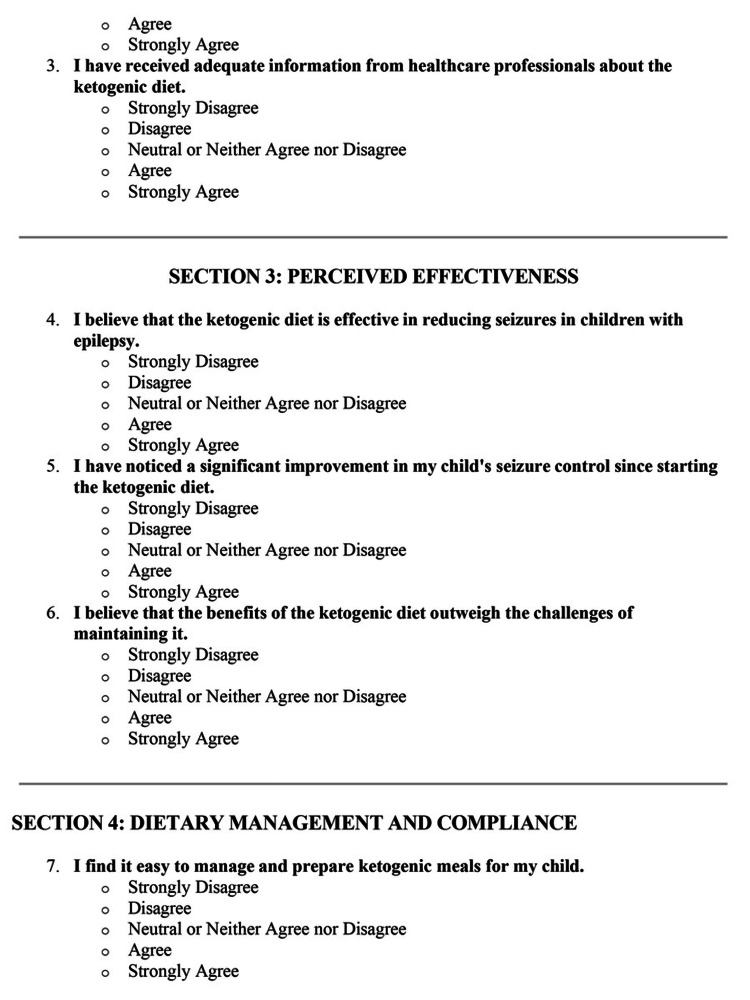
Questionnaire Page 10

**Figure 11:**
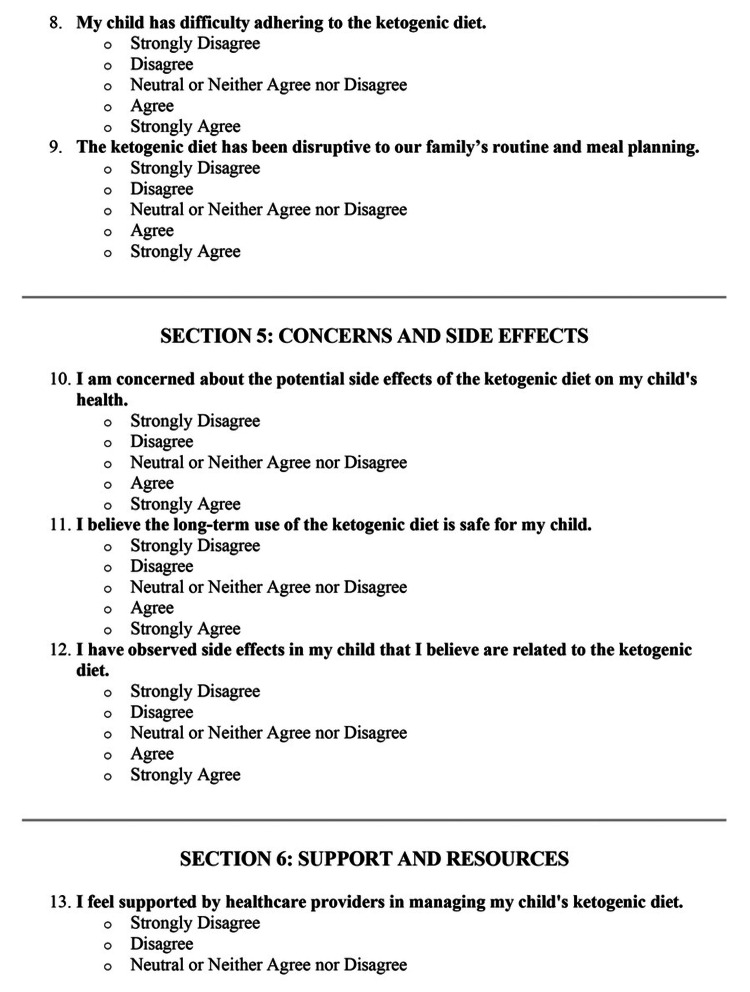
Questionnaire Page 11

**Figure 12:**
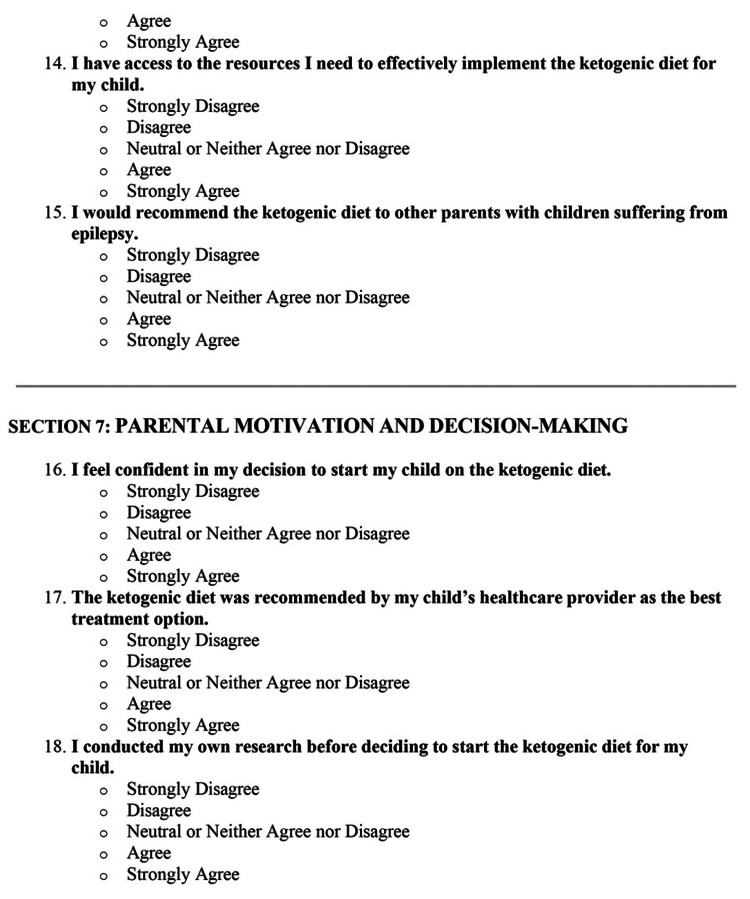
Questionnaire Page 12

**Figure 13:**
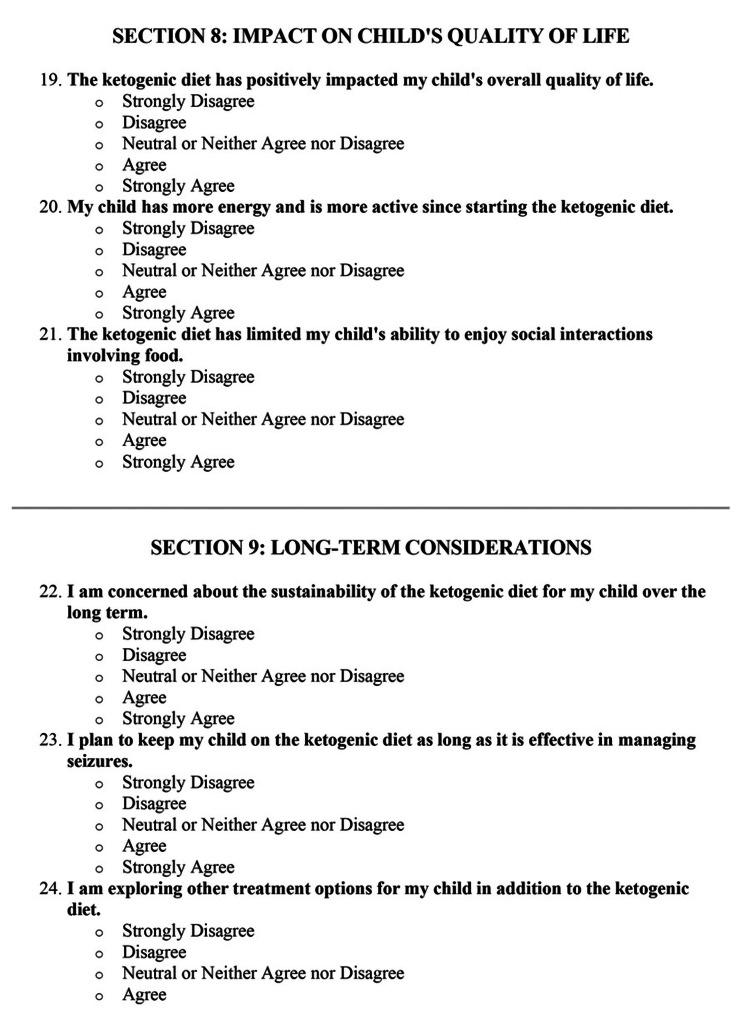
Questionnaire Page 13

**Figure 14:**
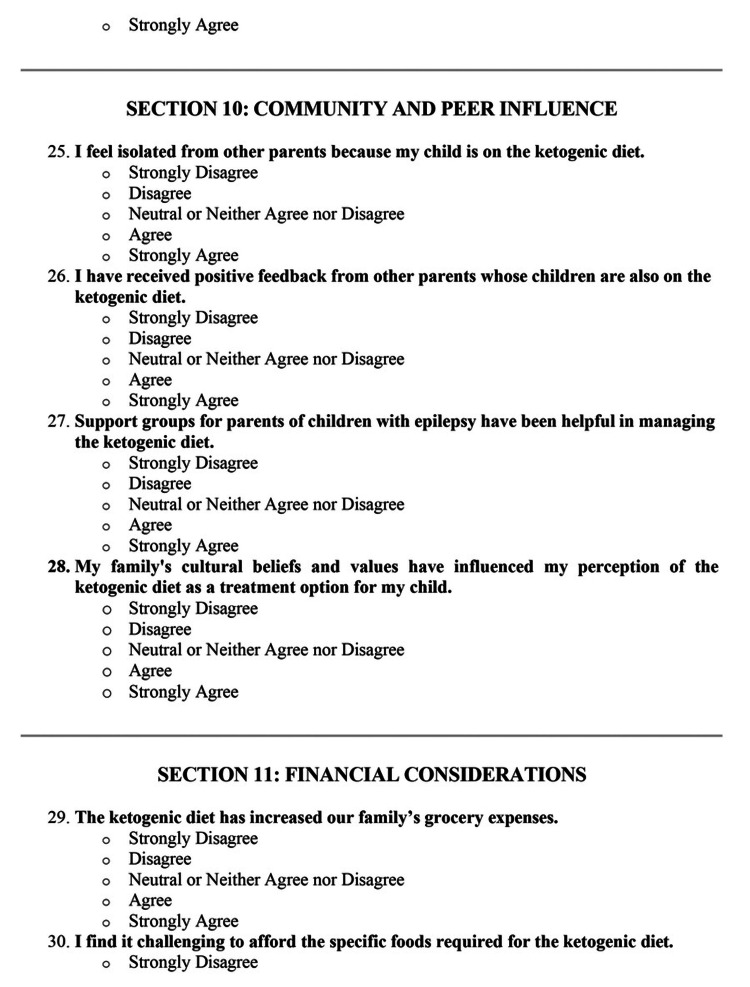
Questionnaire Page 14

**Figure 15:**
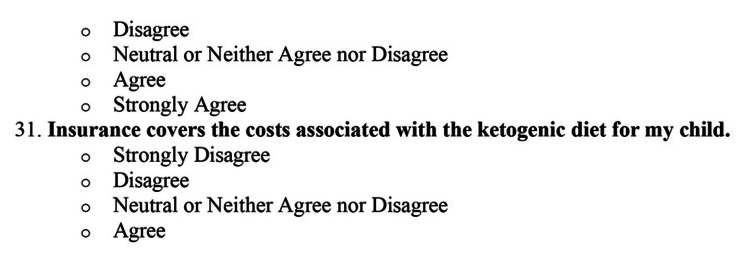
Questionnaire Page 15

## Appendices

The questionnaire used in the present study is shown in Figures 1-15.
